# Book Reviews

**Published:** 1897-05

**Authors:** 


A System of Practical Medicine. By American authors. Edited by Alfred Lee Loomis, M. D., Late Professor of Pathology and Practical Medicine in the New York University, and William Gilman Thompson, M. D., Professor of Materia Medica, Therapeutics and Clinical Medicine in the New York University. To be completed in four imperial octavo volumes, containing from 900 to 1,000 pages each, fully illustrated in colors and in black. Vol. I. Infectious diseases. Just ready. Vol. II. Diseases of the respiratory and circulatory systems, and of the blood and kidneys. In press. Vol. III. Diseases of the digestive system, of the liver, spleen, pancreas and other glands ; gout, rheumatism, diabetes and other constitutional diseases. In active preparation. Vol. IV. Diseases of the nervous system and of the muscles ; diseases of doubtful origin, insolation, Addison's disease, etc. In active preparation. Per volume, cloth, $5.00 ; leather, $6.00 ; half-morocco, $7.00. Philadelphia and New York: Lea Brothers & Co., Publishers.

The fashion of encyclopedic medical literature seems to have become pretty well established. It is a fashion that has its advantages and disadvantages. On the one hand, authors of monographs are enabled to speak from a large experience in that branch of medicine to which they may have devoted special study; on the other, a work constructed on these lines is liable to lack that uniformity and consecutiveness of thought and expression, and withal that absence of repetition that a treatise by a single author is more apt to possess.

This treatise was begun by the late Dr. Alfred L. Loomis, who associated himself with William Gilman Thompson in the editorship of the treatise. Since the death of Dr. Loomis, January 23, 1895, Dr. Thompson has carried on the work as sole editor-in-chief. It is doubtful if any man could have been more appropriately chosen for this office than Dr. Thompson. His longtime intimacy with Dr. Loomis, his thorough knowledge of the latter's methods and plans, and withal his own skill as a physician and eminence as a teacher, pointed him out as one specially fitted to conduct this great task to completion.

In the preparation of this comprehensive system of medicine the editors have been fortunate in the choice of contributors. They have not only selected men of conspicuous eminence in the several departments pertaining to medicine, bacteriology, pathology, state medicine, hygiene, and in several specialities, but they have also been well distributed as to geography, so that the system represents all parts of the United States.

The first volume contains the names of nineteen eminent clinicians and teachers who share authorship in its production and who have presented their several monographs in comprehensive, dignified and concise style, quite in keeping with the necessities of the present day and age. It is devoted to the consideration of infectious diseases, and is undoubtedly the most comprehensive exposition of these maladies that has been published in the United States, if not in the world. There are 950 pages of the treatise proper, besides an index of thirty-five pages, the latter double-columned. There is great economy of expression in these pages, so that they contain more material than is usually found in the same amount of space. References in copious foot-notes abound throughout the volume, enabling the reader to look up a quoted author in his original tongue and publication.

Finally, we may with propriety allude to the mechanical execution of the book. It is printed in heavy-faced type, on well-finished book paper, and bears the artistic finish that characterises every book that issues from Mr. Dornan's well-ordered presses under the supervision of Lea Brothers & Co. Illustrations and charts have been introduced wherever necessary to elucidate the text, some of which are in colors and nearly all of artistic excellence.

We have thought it quite unnecessary to present an analysis of the different subjects treated in this volume. That seems to be a method wholly uncalled for at the present time, the principal object of which would seem to be to display the erudition of the reviewer. A brief description of the scope and make-up of the work is quite sufficient to enable our readers to judge of its merit.

---

LECTURES ON RENAL AND URINARY DISEASES. By ROBERT SAUNDBY, M. D., Edin., F. R. C. P., Lond., Emeritus Senior President of the Royal Medical Society ; Fellow of the Royal Medico-Chirurgical Society ; Professor of Medicine in Mason College, Birmingham, etc., etc. With numerous illustrations. Second edition. Small 8vo, pp. xii.—434. Philadelphia : W. B. Saunders. 1897.

This book represents a union of the author's lectures on Bright's disease published in 1889, and his lectures on diabetes published in 1891, to which he has added a section on miscellaneous renal diseases in order to make the book more complete as a work of reference. Saundby is an interesting writer and is thoroughly familiar with his subject. He is recognised as authority on renal diseases in every quarter of the globe, and this present work, in its enlarged and improved form, will be sought by every physician who must needs supply himself with the latest and best teaching on the subjects with which it treats.

---

OVER THE HOOKAH. The Tales of a Talkative Doctor. By G. FRANK LYDSTON, M. D., Fellow of the Chicago Academy of Medicine, the Southern Surgical and Gynecological Association, and the American Academy of Social and Political Science ; Professor of Criminal Anthropology in the Kent College of Law. Illustrated, from the author's designs, by Mr. C. Everett Johnson. Chicago : Fred Klein Co. 1896.

This book is an interesting grouping of poetry, prose and art, that will serve to entertain many a weary doctor fortunate enough to possess it. The author is well known to the medical profession of the United States as a "prince of good fellows" and as a scientific physician.

We cannot attempt to speak in detail of the stories herein told, but in the main they are of a character that appeals to the intelligent mind, are restful and mirth-provoking, and cannot fail to be acceptable to a profession that gets too little rest and is vastly overworked. We desire to speak in the highest terms of the illustrations, many of which betray high artistic conception in design and some are exceedingly well executed. On the whole, the book presents an excellent appearance, yet it lacks a finish that Putnam or Dornan would have given it.

---

Autoscopy of the Larynx and the Trachea. (Direct examination without mirror.) By Alfred Kirstein, M. D., Berlin. Authorised translation (altered, enlarged and revised by the author,) by Max Thorner, A. M., M. D., Cincinnati, O., Professor of Clinical Laryngology and Etiology, Cincinnati College of Medicine and Surgery; Laryngologist and Aurist, Cincinnati Hospital, etc. With twelve illustrations. One volume, 12mo, pp. xi.—68. Philadelphia: The F. A. Davis Co., Publishers. 1897.

In this little book will be found all that is known in regard to this somewhat novel method employed in the diagnosis and treatment of diseases of that portion of the upper air-tract called the larynx and trachea. Kirstein is an enthusiast on the subject of making direct examination and treatment of the larynx and trachea without the aid of a mirror, and has devised a number of instruments with that end in view. The translator, who has done his part of the work well, is also a believer in this method. We are inclined to the belief that its practicability is limited; nevertheless, it is an additional means of diagnosis and treatment that should be appealed to in suitable cases.

---

Artificial Anesthesia. A Manual of Anesthetic Agents and Their Employment in the Treatment of Disease. By Lawrence Turnbull, M. D., Ph. G., Aural Surgeon to the Jefferson Medical College Hospital, Philadelphia; late Honorary President to the Otological Subsection of the British Medical Association and of the Section of Laryngology and Otology of the American Medical Association. Fourth edition, revised and enlarged, with illustrations. Duodecimo, pp. 550. Philadelphia: P. Blakiston, Son & Co., 1012 Walnut street. 1896.

It is near twenty years since the first edition of this work was published. It had its origin in a report for a medical society and was subsequently enlarged to book-form to meet a demand on the part of the profession for a handbook on the administration of anesthetics. It is six years since the third edition was sent out. This having become exhausted, the publishers appropriately determined to issue this fourth edition in the year 1896, which was the fiftieth anniversary of the discovery and introduction of ether. This, the author says in his preface, is the one systemic anesthetic which has proved during all these years the most available and freest from danger.

This manual contains full directions for the administration of the several anesthetics, a description of the various inhalers, and methods for resuscitation in case of threatened danger, to which is added a short chapter on the legal responsibility of physicians in the administration of anesthetics. Local anesthesia is fully discussed and formulæ for local anesthetics are given. It is a book that every surgeon should possess and every anesthetist study.

---

TRANSACTIONS OF THE AMERICAN SURGICAL ASSOCIATION. Volume XIV. Edited by DE FOREST WILLARD, A. M., M. D., Ph. D., Recorder of the Association. Printed for the association. For sale by Wm. J. Dornan, Philadelphia. 1896.

This is one of the largest and best volumes yet published by this distinguished association. It contains 742 pages, including many illustrations of value. The papers and discussions of this association are noted for their scientific excellence. At this meeting tuberculosis formed the central feature of interest and was presented in papers by Drs. N. Senn, A. Vander Veer, Robert Abbe, George R. Fowler, De Forest Willard, J. McFadden Gaston and George W. Gay. A paper on Susceptibility and Immunity, with special reference to surgical cases, was presented by Dr. Roswell Park, of Buffalo. An exhaustive paper on the Surgical peculiarities of the negro, was presented by Dr. Rudolph Matas, of New Orleans. The volume is executed in Mr. Dornan's best style, though we would enjoy it better if the edges were cut.

---

THE MICROSCOPE AND MICROSCOPICAL METHODS. By SIMON HENRY GAGE, Professor of Microscopy, Histology and Embryology in Cornell University and the New York State Veterinary College, Ithaca, N. Y. Sixth edition, rewritten, greatly enlarged and illustrated by 165 figures in the text. Ithaca, N. Y.: Comstock Publishing Co. 1896.

The appearance of a new edition of Prof. Gage's admirable work on microscopy and histology is evidence of the ever-gaining popularity of this treatise. Started in a humble way it has grown into a volume of some proportion and has kept pace with the latest thought and investigations in the science of which it treats. The figures have been increased from 103, in Volume V., to 165, including the device described by Dr. Krauss, of Buffalo, for marking objectives on a revolving nose-piece. The chapters on photomicrography have been revised and extended and are now quite complete.

The new edition deserves even more of the kind things said of previous editions and will, no doubt, become exhausted in a short time.

PRINCIPLES OR GUIDES FOR A BETTER SELECTION OR CLASSIFICATION OF CONSUMPTIVES, amenable to high altitude treatment and to the selection of patients who may be more successfully treated in the environment to which they were accustomed previous to their illness. By A. EDGAR TUSSEY, M. D., Adjunct Professor of Diseases of the Chest in the Philadelphia Polyclinic and School for Graduates in Medicine and Consulting Physician to the central branch of the Y. M. C. A. Gymnasium. Fifteenth and Chestnut streets. Philadelphia: P. Blakiston, Son & Co., 1012 Walnut street. 1896.

The treatment of consumption in high altitude is set forth in this book with considerable argumentative force. The style of the author is somewhat flowery, but he is admittedly a conscientious student of his subject, and what he has to say is worthy of consideration. The tendency now is toward the isolation of consumptive patients, and experience has shown that the high altitude of the Adirondack region exercises a beneficial and sometimes even a curative influence upon the malady. To those interested in a study of the subject from the standpoint of the author, his book will be regarded in acceptable light.

---

HOW TO LIVE LONGER AND WHY WE DO NOT LIVE LONGER. By J. R. HAYES, M. D., Medical Examiner Bureau of Pensions, Department of the Interior, Washington, D. C.; late Surgeon United States Volunteers; late Medical Director Department of the Potomac, G. A. R. Philadelphia: J. B. Lippincott Co. 1897.

The unusual title here offered will attract many a reader who probably would pay very little attention to its subject-matter in ordinary dress and with a commonplace name. Dr. Hayes portrays a ripe scholarship and an easy style in dealing with his subject. He teaches us how to let good digestion wait on appetite, but particularly to be commended are his chapters on alcohol and tobacco. It is a little volume that may be well allowed to grace everybody's library and its teachings are such as to commend it in a special way to the young.

---

THE YEAR-BOOK OF TREATMENT FOR 1897. A critical review for practitioners of medicine and surgery. Crown octavo, 488 pages. Philadelphia and New York: Lea Brothers & Co. 1897.

This annual visitor is always welcomed by the profession because, first, of its convenient size and, second, because of its form, general arrangement and methodical selection of its material. It is a critical condensation of the progress made during 1896 in all the branches of medicine and surgery that have practical interest to the physician. Each branch is presented with sufficient detail to make it of practical use as an index, and it is well classified so that it is convenient for reference in emergencies. The promptness with which the book appears, its thoroughly practical character and its inexpensive price ($1.50) appear to us points of excellence that should commend it to the busy practiser of medicine.

A MANUAL OF THE PRACTICE OF MEDICINE, prepared especially for students. By A. A. STEVENS, A. M., M. D., Lecturer on Terminology and Instructor in Physical Diagnosis in the University of Pennsylvania; Demonstrator of Pathology in the Woman's Medical College of Pennsylvania; Physician to St. Agnes's Hospital, to the out-patient department of the Episcopal Hospital and to the Southeastern Dispensary, Philadelphia. Fourth edition, revised and enlarged. Illustrated. Pp. xviii.—511. Philadelphia: W. B. Saunders, 925 Walnut street. 1896.

The frequency with which new editions of this manual are demanded bespeaks its popularity. It is an excellent condensation of the essentials of medical practice for the student, and may be found also an excellent reminder for the busy physician. This edition has been revised and additions have been made with a view to bring it forward to the immediate present. It is announced that an Italian edition is now in progress, which further accentuates the favor with which the work is received.

---

ELEMENTARY BANDAGING AND SURGICAL DRESSING, with directions concerning the immediate treatment of cases of emergency for the use of dressers and nurses. By WALTER PYE, F. R. C. S., late Surgeon to St. Mary's Hospital. Revised and in part rewritten by G. Bellingham Smith, F. R. C. S., Surgical Registrar, Guy's Hospital. 16mo, pp. 218. Seventh edition. Philadelphia: W. B. Saunders, 925 Walnut street. 1897.

This book has apparently been one of the popular little manuals, for already six editions have been exhausted in eleven years. The present edition has been revised to meet recent advances in surgical practice and this, too, has necessitated some additions. The progress made in the dressing of wounds has been noted by the rewriting of the chapter on that subject. Nurses in surgical wards will find this a very useful guide.

